# Developing an Integrated Toolbox for Raman Spectral Analysis with Both Artificial Neural Networks and Machine Learning Algorithms

**DOI:** 10.3390/molecules31040666

**Published:** 2026-02-14

**Authors:** Xiangtao Kong, Jie Xu, Guodi Fan, Zixuan Zhang, Qidong Liu, Haorui An, Shuang Wang

**Affiliations:** 1 Institute of Photonics and Photon-Technology, Northwest University, Xi’an 710069, China; 2 College of Life Science, Northwest University, Xi’an 710069, China

**Keywords:** quantitative analysis, machine learning, neural network, AI-Raman, Raman spectra

## Abstract

Based on its rich information of chemical specificity, Raman spectroscopy has been widely applied for in vivo biomedical investigations. For extracting quantitative information of target constitution, it is imperative to establish a robust model for unveiling the relationship between spectral features with/without priori references. By integrating a variety of traditional machine learning and artificial neural network algorithms, an integrated Raman spectra analysis toolbox (AI-Assisted Raman Spectra Analysis Toolbox [AI-Raman] V 1.0) was developed for spectral processing, model training, and regression analysis by using MATLAB R2024a. Besides the utilization of back propagation artificial neural network and convolutional neural network algorithms, classical machine learning algorithms, such as partial least squares regression and support vector regression, were also compacted as the supporting functions of presented toolbox. A spectral dataset obtained from nailfold from different subjects was utilized to evaluated the feasibility and performance of the developed software, which demonstrated that the analysis software can predict glucose concentrations by in vivo Raman spectral measurement. With a friendly graphics interface, the analytical model can be customized and optimized for accomplishing the desired objectives, which will benefit many Raman-based inventions, especially for biomedical transformations.

## 1. Introduction

Vibrational spectroscopy comprises label-free techniques that reveal electronic changes in the internal vibrational energy levels of biomolecules. As one of the main techniques used to assess vibrational modes, Raman spectroscopy is based on the inelastic scattering effects between photons and molecules [1]. Raman spectral signals come from both Stokes (photons with lower energy are emitted) or anti-Stokes (photons with higher energy are emitted) emission [2]. Since anti-Stokes signals are relatively weak and usually depend on the molecular gathering on high-energy states, the Stokes signals are usually recorded as Raman spectra, which is also called spontaneous Raman spectroscopy (RS). Based on the rich information of biomolecular specificity provided by RS, it facilitates numerous novel applications in biochemical and biomedical investigations because of its minimal sample preparation, label-free, non-destructive nature, and substantially accurate results.

However, Raman spectroscopy faces inherent limitations in biomedical applications that hinder its practical clinical transformation. Two major challenges are fluorescence interference and matrix effects. Endogenous fluorophores in biological samples (e.g., proteins, flavins, and porphyrins) often produce strong fluorescence signals that overlap with weak Raman scattering, masking the target spectral features [3]. Additionally, matrix effects, arising from the complex and heterogeneous composition of biological matrices (e.g., overlapping signals from water, lipids, and proteins in tissue), can further distort the Raman peaks of target analytes (e.g., low-concentration glucose) and reduce quantitative accuracy [4]. To mitigate these issues, researchers have developed various strategies, including surface-enhanced Raman scattering (SERS) to amplify weak Raman signals, fluorescence-quenching agents to suppress background fluorescence, advanced baseline correction algorithms (e.g., polynomial fitting and adaptive iteratively reweighted penalized least squares), and multivariate data analysis techniques to separate target signals from matrix interference [5].

Meanwhile, instrument advances have accelerated the application of Raman spectroscopy in biomedicine, with probe-based Raman systems emerging as a key trend [6]. These miniaturized, flexible probe systems enable in situ, real-time Raman measurements in hard-to-reach or delicate biological sites (e.g., internal organs, nailfold, and mucosal tissues), overcoming the limitations of traditional bench-top Raman spectrometers that require sample extraction and off-site analysis. Recent advancements in probe design (e.g., fiber-optic Raman probes with high signal collection efficiency) and detector technology have further improved the sensitivity and spatial resolution of these systems, facilitating their integration into clinical workflows [7]. Despite these advancements, the analysis of Raman spectral data—especially from complex biomedical samples—remains fragmented, with no unified platform to integrate preprocessing, model training, and evaluation, highlighting the need for the toolbox developed in this study.

Before understanding and interpreting bio-spectral data, preprocessing is usually applied to the spectral data for reducing chemically irrelevant variations. The main goal of preprocessing is to improve the identifiability of chemical features in spectral data, which is essential for correcting physical interferences, such as light scattering due to different particle sizes, random noise from instruments, and substrate contributions. Practically, preprocessing procedures should be operated with caution and in a logical order based on previous experiences, since they would carry the risk of generating correlations in the noise structure which would bring negative impacts on the following analysis tasks. Many algorithms have been developed and introduced for achieving optimal or close to optimal results based on the specific experimental objectives [8,9,10].

Moreover, by adapting mathematical or statistical methods, diversified algorithms for spectral mixtures have been developed to either categorize the spectra to distinct classes (classification) or try to extract quantitative information (regression). Designing an optimal model is challenging for either classification or regression tasks. Based on the correlation between spectral features and chemical constituents, some multivariate algorithms have been developed and employed to identify the similarities or dissimilarities between samples or assess the actual chemical concentration, such as principal component analysis (PCA) [11], linear discriminant analysis (LDA) [12], quadratic discriminant analysis (QDA) [13], partial least squares (PLS) [11], discriminant analysis (PLS-DA) [11,14], and so on. In most of the classification algorithms, the discriminant tasks were completed by a combination of feature extraction or selection methods, followed by modeling chosen techniques. For improving the convenience of pre-processing operations and the following analysis steps, many commercial programs and open-source toolboxes have also been developed by integrating the versatile functions for diversified application requirements, among which an integrated Raman spectral analysis program (NWUSA [15]) was developed by our group for general analytical objectives.

As an alternative, artificial neural networks (ANNs) are capable of extracting chemically meaningful results from the acquired Raman spectral data, such as a convolutional neural network (CNN), multi-Layer Perceptron (MLP) trained with a backpropagation algorithm, and so on. Genetic algorithms (GAs), as an evolutionary optimization tool, can also be used to optimize hyperparameters of the proposed models (e.g., MLPs trained with backpropagation and CNNs) but are not classified as artificial neural networks. Principally, ANNs learn the patterns of a hyperspectral dataset and recognize potential information from the input data for accurately creating linear and nonlinear relationships between bio-spectra and chemical constituents. Compared to traditional multivariate algorithms, ANNs can mitigate the risk of overfitting under appropriate model design, sufficient data support, or regularization strategies, and exhibit good generalization ability when hyperparameters are properly tuned [16,17]. Due to this, many studies have adapted ANN algorithms for classification or quantitative analysis on vibrational spectroscopic data. A CNN model based on clinical data was introduced to effectively distinguish between cancerous and normal pancreatic tissue [18]. An artificial neural network was also designed to accurately differentiate the type 2 diabetes group from the healthy control group with an accuracy of 88.9–90.9% [19]. By training a CNN to identify 30 common bacterial pathogens, high accuracy can be achieved even on low signal-to-noise ratio Raman spectra [20].

In order to fulfill the growing needs for executing ANN-based Raman spectral analysis models, we introduced a home-made open-source toolbox by integrating traditional preprocessing and ANN algorithms into an easy-to-use but logical interface. To illustrate the toolbox’s practical utility, we take non-invasive blood glucose prediction as a typical application by using our published dataset [21,22]. Researchers can use this toolbox to process nailfold Raman spectra, train regression models, and achieve quantitative glucose prediction—all through an intuitive graphical interface, without the need for complex programming. Following our previous works [15,23], this integrated toolbox, named AI-Assisted Raman Spectra Analysis Toolbox (AI-Raman), was developed for handling complex regression tasks where multiple spectral variables were comprehensively utilized to quantify unknown spectral information with/without priori references. Besides the realization of MLP (trained with backpropagation) and CNN algorithms, classical machine learning algorithms, like partial least squares regression (PLSR) and support vector regression (SVR), were also added as additional supporting functions. The trained and tested models could be saved and utilized for interpreting RS more conveniently and accurately in some specific applications, especially for biomedical investigations.

## 2. Results

**Figure 1:**
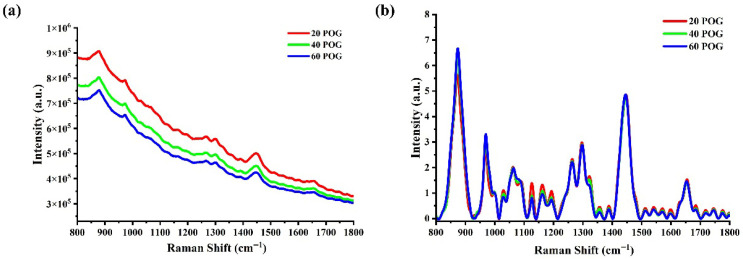
Representative raw and preprocessed Raman spectra of human wrist skin. (**a**) The original in vivo Raman spectra were respectively obtained from the blood vessels in the right wrist of human subjects post oral glucose (POG) at 20 (20 POG), 40 (40 POG), and 60 (60 POG) minutes. (**b**) The preprocessed Raman spectra (after cosmic ray removal, EMSC background subtraction, and Savitzky–Golay smoothing) corresponding to the raw spectra in Panel (**a**).

For testing of the software, the Raman spectral data of human nailfold collected from different batches were saved in a file in *. mat format and the dataset, comprising a total of 75 Raman spectra, was used for model training and prediction of blood glucose concentration. All the collected Raman spectra were preprocessed according to the above-mentioned steps and methods. To demonstrate the effect of preprocessing, representative raw and processed spectra are shown in Figure 1. Raw spectra are dominated by a strong fluorescence background (Figure 1a), which masks the weak glucose-specific signal. After preprocessing, the background is eliminated and the glucose peak at 1125 cm^−1^ is clearly resolved (Figure 1b). The preprocessed dataset was randomly segmented and 10 of the preprocessed spectra were selected as the prediction set for quantitative regression, while 80% of the remaining data were categorized as the training set and 20% as the test set.

**Figure 2:**
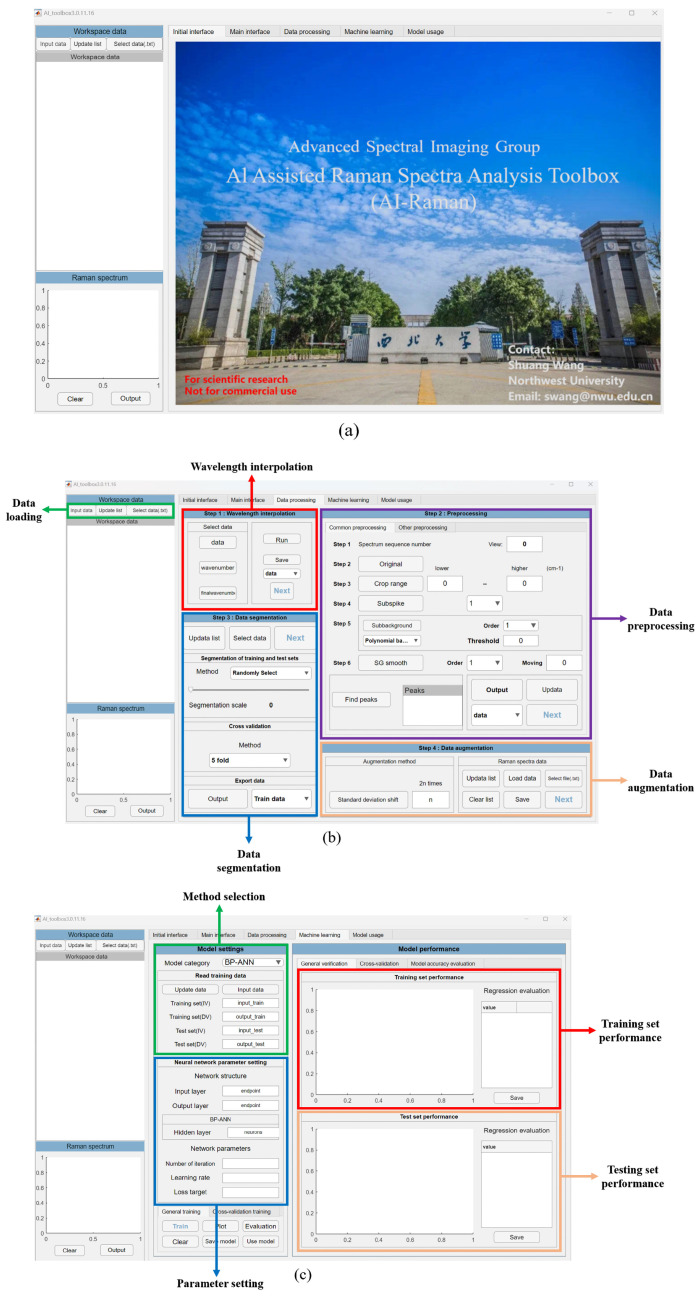

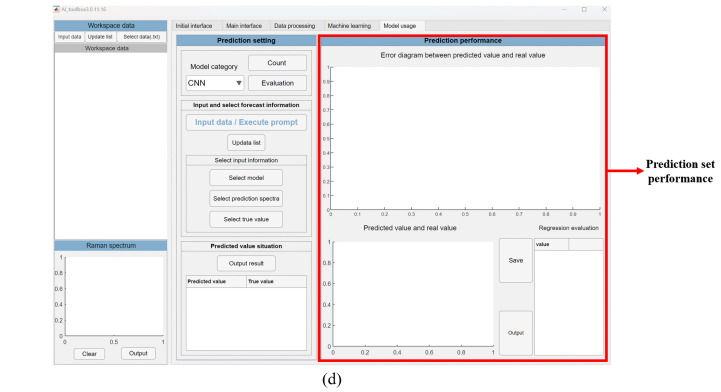
The interface of AI-Assisted Raman Spectra Analysis Toolbox (AI-Raman). (**a**) The initial interface of AI-Raman, in which the Chinese characters stand for Northwest University. (**b**) The interface of data processing module with the functions of performance data preprocessing and data augmentation. It also presents the preprocessed spectrum in the Raman spectrum coordinate region. (**c**) The interface of machine learning module, providing the MLP (trained with backpropagation), CNN, PLSR, and SVR algorithms for analysis tasks. (**d**) The interface of the model usage module.

**Figure 3:**
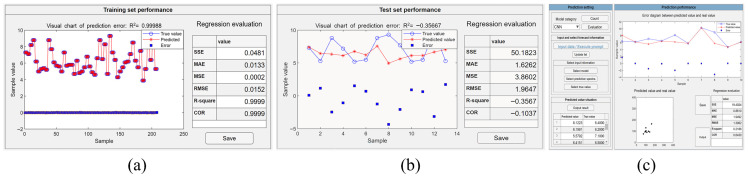
The example output of the regression model constructed by the CNN algorithm with different datasets. (**a**) The performance evaluation on the constructed model with training dataset. (**b**) The performance evaluation on the constructed model with test dataset. (**c**) The performance evaluation on the constructed model with the prediction dataset.

Before applying the CNN algorithm, the dataset was augmented to four times its original size with the standard deviation shift method in the interface shown in Figure 2b. The necessary training parameters were set during the training process in the interface shown in Figure 2c, in which 1000 iterations and a learning rate of 0.001 were applied. When the model training started, an indicator interface was displayed on the screen, representing the progress of the task. During the training process, users could monitor the proceedings in real time and pause it at any time at their will. When the training was completed or manually terminated, error plots and regression evaluation tables of the training and test sets were generated and displayed as shown in Figure 3a and Figure 3b, respectively. Based on the regression evaluation tables for the training and test sets, models with smaller errors could be saved to perform quantitative analysis on the prediction set. The performance of the model on the prediction set is shown in Figure 3c. The RMSE of the trained CNN model on the prediction set is 1.3962 mmol/L with an R^2^ of 0.2186. This indicates that the model has a large error on the prediction set and the model has limited generalization ability. This may be due to the low signal-to-noise ratio of the data, which caused the model to capture some noise in the training data during the training process, thus affecting the model’s performance on the prediction set.

**Figure 4:**
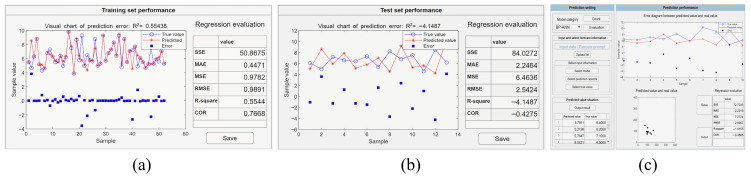
The example output of the regression model constructed by the MLP trained with a backpropagation algorithm with different datasets. (**a**) The performance evaluation on the constructed model with training dataset. (**b**) The performance evaluation on the constructed model with test dataset. (**c**) The performance evaluation on the constructed model with the prediction dataset.

A MLP trained with backpropagation is a fully connected neural network without a specialized feature extraction structure, where each neuron is connected to all the neurons in the previous layer, with their inputs weighted and summed. When using a MLP trained with backpropagation in the software, the input layer has the same number of nodes as the number of spectral features, and the output layer has only one neuron for the blood glucose concentration. The number of neurons in the hidden layer could be set manually according to the task to improve the performance of the network. For enhancing the generalization ability of the model, an early stopping method was incorporated in the software by setting the loss objective, so that training stops automatically when the training error reaches or falls below this objective. The initial parameters of training procedure were set as follows: the number of neurons in the hidden layer was set to 4, the number of learning to 100, the learning rate to 0.001, and the loss target was set to 0.0001. After updating all of related parameters shown in Figure 2c, the training model would start automatically without manual intervention. The performance of the training set and the test set can be evaluated by clicking the evaluation button, as shown in Figure 4a and Figure 4b, respectively. After achieving the satisfactory trained model, the prediction set data can be inputted into the model usage module, and its output results and achieved accuracy will be displayed in the module, as shown in Figure 4c. The RMSE of this MLP trained with a backpropagation model on the prediction set is 2.6967 mmol/L and R^2^ is −1.9155.

**Figure 5:**
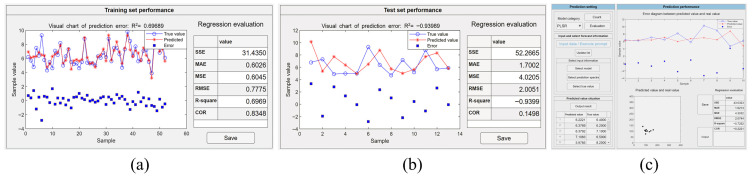
The example output of the regression model constructed by the PLSR algorithm with different datasets. (**a**) The performance evaluation on the constructed model with training dataset. (**b**) The performance evaluation on the constructed model with test dataset. (**c**) The performance evaluation on the constructed model with the prediction dataset.

**Figure 6:**
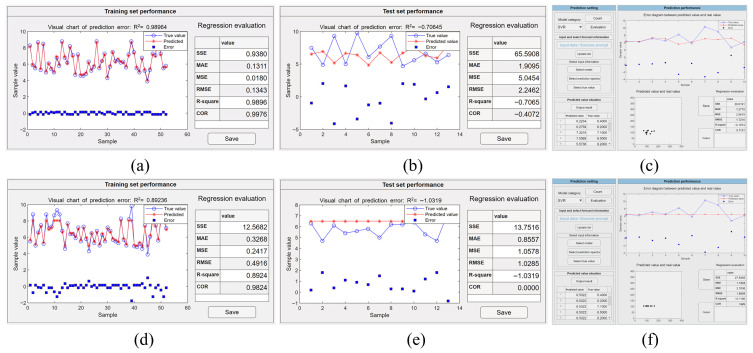
The example output of the regression model constructed by the SVR algorithm with different datasets and kernels. (**a**) The performance evaluation on the constructed SVR (linear kernel) model with training dataset. (**b**) The performance evaluation on the constructed SVR (linear kernel) model with test dataset. (**c**) The performance evaluation on the constructed SVR (linear kernel) model with the prediction dataset. (**d**) The performance evaluation on the constructed SVR (Gaussian kernel) model with training dataset. (**e**) The performance evaluation on the constructed SVR (Gaussian kernel) model with test dataset. (**f**) The performance evaluation on the constructed SVR (Gaussian kernel) model with the prediction dataset.

Compared to a CNN and MLP trained with backpropagation implementations, it is much easier to facilitate PLSR and SVR algorithms in the presented toolbox. When applying PLSR analysis, it is sufficient to select the algorithm in the interface shown in Figure 2c and train it directly. It is a critical step to determine the appropriate number of latent variables, since too many variables may lead to model overfitting, while too few may result in underfitting. During the execution of the program, the software will set the number of latent variables with the smallest average MSE value based on the cross-validation results, and then construct the regression model using the training set data and complete the regression prediction for the test set data. The output accuracy of the training and test datasets along with the regression evaluation results can be identified respectively in the regression evaluation column, as shown in Figure 5a,b. After saving the trained model, the data in the prediction set can be input into the usage module and the performance of the prediction set can be identified, as in Figure 5c. Operators can also choose a SVR algorithm by selecting the corresponding function in model selection (Figure 2c). Subsequently, an interface will appear for choosing the kernel function for specific analytical tasks. It includes a grid search algorithm to automatically search for the optimal learning parameters. Although the grid search algorithm is more time-consuming, it spares inexperienced users from manually adjusting parameters and finds more appropriate ones automatically. The achieved results of example models using the linear and Gaussian kernel functions on the training set are shown in Figure 6a,d and on the test set in Figure 6b,e. After saving both models, the achieved results of the prediction set data in the model usage module are shown in Figure 6c and Figure 6f, respectively.

When using neural network-based deep learning algorithms, the crucial factors include the structure of the model, learning parameters, and data dimensions. A CNN automatically performs feature extraction through its convolutional layers and uses pooling layers to reduce the data dimensions, offering inherent advantages in processing high-dimensional data [24,25]. Nevertheless, training a CNN model requires a large amount of data and meticulous tuning of numerous hyperparameters, often necessitating extensive experimentation and debugging. In contrast, the structure of a MLP (trained with backpropagation) is relatively simple, lacking complex convolutional and pooling layers, which allows for faster training speeds. However, a MLP (trained with backpropagation) lacks the ability to automatically extract and learn features, typically requiring manual feature extraction [26]. This increases the complexity and difficulty of model construction, especially when dealing with complex data. The prediction results of CNN and MLP (trained with backpropagation) models for the same dataset with multiple samples are presented in Figure 3b and Figure 4b, respectively, with the values of RMSE of 1.9647 mmol/L and 2.5424 mmol/L and the values of R^2^ of −0.3567 and −4.1487, respectively. The RMSE value of the CNN is less than the MLP (trained with backpropagation) and R^2^ is greater than the MLP (trained with backpropagation). The results demonstrate that, for the preprocessed Raman spectral data without feature extraction, the CNN model exhibits superior performance compared to the MLP (trained with backpropagation) model.

The SVR algorithm skillfully employs kernel functions to address both linear and nonlinear problems [27,28] and can exhibit good robustness to noisy data through the use of relaxation variables [29]. For data with irregular or abnormal values, SVR can yield relatively stable results. However, the SVR algorithm involves many hyperparameters, increasing computational complexity. In addition, improper setting of these hyperparameters may increase the risk of model overfitting. As shown in Figure 6a,d, the R^2^ values of the linear and Gaussian kernel function SVR models in the training set are 0.9896 and 0.8924, respectively, with small RMSE values. In the test set, the R^2^ values are −0.7065 and −1.0319 and the RMSE values are 2.2462 mmol/L and 1.0285 mmol/L, respectively, as shown in Figure 6b,e, which indicates an overfitting problem. This due to the fact that PLSR is relatively suitable for dealing with high-dimensional collinear data problems [30], but may be unable to deal with nonlinear problems. Additionally, the selection of the number of components in PLSR often requires empirical determination or cross-validation. If the number of components is too large, the model may become too complex, capturing noise from the training data. Although the optimal number of potential components can be determined by cross-validation in this software and used for subsequent model training, the performance of the model is still unsatisfactory. As shown in Figure 5b, the R^2^ value of PLSR is −0.9399 and the RMSE is 2.0051 mmol/L. The PLSR error is larger than that of the SVR model constructed with a Gaussian kernel and smaller than that of the SVR model constructed with a linear kernel.

## 3. Discussion

Computational efficiency is critical for the AI-Raman toolbox’s applicability to routine laboratory research, where rapid data analysis is often required. All models were tested on a standard desktop computer (Intel Core i7-12700 CPU, 16 GB RAM) to reflect common hardware access. Classical machine learning models (PLSR/SVR) offer the fastest turnaround (~30 s per model), making them ideal for high-throughput preliminary screening of multiple datasets. The MLP’s ~45 s duration balances complexity and efficiency, suitable for users seeking nonlinear modeling without deep learning overhead. The CNN’s ~60 s duration (with data augmentation) is acceptable for deep learning tasks on CPU-only hardware, avoiding reliance on specialized GPUs and aligning with the toolbox’s goal of broad accessibility. Notably, computational durations scale moderately with dataset size: doubling the sample count (from 300 to 600) increased CNN training time to ~90 s, while PLSR/SVR durations rose to ~45 s—still within practical timeframes for most biomedical research scenarios.

For Raman spectral data without artificial feature extraction, CNNs typically perform better than MLPs (trained with backpropagation), PLSR, and SVR, as these latter algorithms lack strong feature extraction capabilities [31]. As shown in Figure 3b, Figure 4b, Figure 5b and Figure 6b,e, the RMSE values of the CNN, MLP (trained with backpropagation), PLSR, SVR (linear kernel), and SVR (Gaussian kernel) models on the test set are 1.9647 mmol/L, 2.5424 mmol/L, 2.0051 mmol/L, 2.2462 mmol/L, and 1.0285 mmol/L, respectively. Although the CNN does not require time-consuming steps to find the best data preprocessing and feature extraction methods, it does involve pre-determined numerous inherent parameters, such as layer size, kernel size, and step size. The selection of parameters for different datasets can result in significant variations in outcomes, which induce a complex procedure of parameter adjustment. Conversely, PLSR and SVR need fewer input parameters, making it easier to achieve optimal conditions. When data have undergone manual preprocessing and feature extraction, the advantages of a CNN are reduced and the accuracy of PLSR, SVR, and most other algorithms is significantly improved.

The quantitative identification of the corresponding chemicals by Raman spectroscopy is a relatively complex process, which first requires data preprocessing, then model constructing and training, as well as the final evaluation. Therefore, we developed AI-Assisted Raman Spectra Analysis Toolbox based on MATLAB R2024a to perform quantitative analysis of corresponding compounds with the acquired spectra. The software was mainly developed with three ingredients; namely, data processing, machine learning, and model usage. In the data processing module, we provide the necessary data preprocessing function, and incorporates a data augmentation function with a consideration that algorithms or tasks may require a large amount of data. In the machine learning module, four machine learning algorithms, including a MLP trained with backpropagation, a convolutional neural network (CNN), partial least squares regression (PLSR), and support vector regression (SVR), are provided for different study objectives. After statistically evaluating the data from test and training datasets, the well-trained models can be saved for further quantitative analysis of the corresponding compounds. The functionality of the model usage module makes the software more flexible for further quantitative predicting tasks with other batches of data.

Model interpretability is a pivotal requirement for biomedical applications, as it validates the chemical rationality of predictions and fosters stakeholder trust. In the presented AI-Raman toolbox, PLSR leverages regression coefficients and loading vectors to directly link spectral bands to target variables. SVR supports linear coefficient analysis (linear kernel) or SHAP-based decomposition (Gaussian kernel). The CNN/MLP trained with backpropagation can be augmented with post hoc techniques like Grad-CAM and LRP to visualize and quantify critical spectral regions. Future versions of the toolbox will integrate these interpretability modules as core functions—users will be able to generate relevance maps, coefficient plots, and spectral contribution analyses with one click, tailored to the selected model. This extension will address the current limitations and strengthen the toolbox’s applicability in regulated biomedical scenarios (e.g., clinical diagnosis and therapeutic monitoring).

The negative R^2^ values and large RMSEs observed in this study should be interpreted as reflections of data constraints (small sample size and high in vivo spectral noise) rather than evidence of model or software failure. The primary goal of presenting these results is to validate the toolbox’s functional feasibility—specifically, its ability to execute end-to-end regression workflows (preprocessing → training → evaluation → prediction) for diverse algorithms—rather than to achieve state-of-the-art predictive performance. For users working with larger, higher-quality datasets (e.g., ≥500 spectra with high signal-to-noise ratios), the toolbox’s integrated models are expected to yield significantly improved R^2^ and RMSE values, as demonstrated by preliminary tests with simulated high-quality spectral data.

It is important to clarify that the glucose prediction results reported herein should not be directly compared with state-of-the-art Raman-based glucose monitoring studies [32]. Existing high-performance studies typically rely on large-scale datasets (≥500 samples), specialized preprocessing pipelines (e.g., multivariate curve resolution for background separation), and customized model optimization—conditions not prioritized in the current work. The core goal of this study is to validate the AI-Raman toolbox’s functional feasibility (e.g., data loading, multi-algorithm integration, and end-to-end regression) rather than pursue superior predictive performance. For users aiming to replicate or exceed existing glucose prediction results, the toolbox provides a foundational platform that can be extended with large datasets and advanced preprocessing/model tuning.

In this work, we do a preliminary demonstration on the usage of the software by analyzing the blood glucose concentration with in vivo Raman spectra from human nailfolds. Through the analysis of machine learning results and regression index evaluation, the machine learning algorithms incorporated in the software system still have much room for improvement, especially for the overfitting problem, which needs further processing of the data and improving the structure of the neural network. Practically, we need to improve the signal-to-noise ratio of spectral data, select appropriate methods for data preprocessing, and select the best learning parameters through cross-validation and other means to achieve a best model, so as to promote the application of quantitative analysis of Raman spectral data more than biomedical studies and applications.

## 4. Methods

### 4.1. Software Architecture

**Figure 7:**
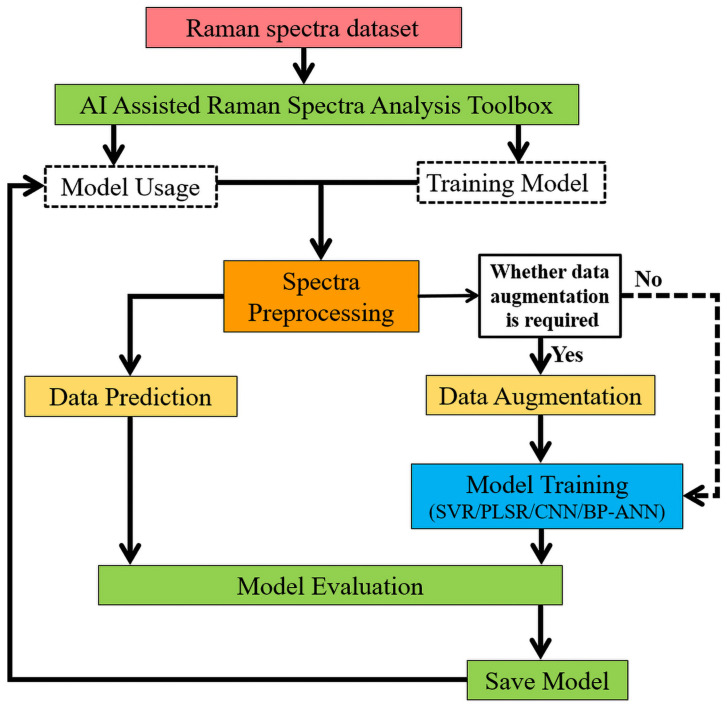
The flow chart of AI-Assisted Raman Spectra Analysis Toolbox (AI-Raman).

As shown in Figure 7, the general implementation of our proposed toolbox mainly included two processes, described as model training and model usage. For model training, the obtained spectral datasets were preprocessed firstly, and then the data augmentation was optionally selected based on the data size needed for the specific algorithm. After that, the CNN, MLP trained with backpropagation, SVR, and PLSR algorithms can be selected to construct regression model for completing the desired quantitative analysis tasks. The achieved accuracy and performance of the constructed model will be evaluated by statistical parameters, including the sum of squares due to error (SSE), mean squared error (MSE), root mean squared error (RMSE), mean absolute error (MAE), coefficient of determination (R^2^), and Pearson correlation coefficient (COR). When the constructed model exhibits satisfactory performance and accuracy, it will be saved and retained for future model usage purposes. During model utilization, the preprocessed dataset for prediction must be loaded for completing the entire regression task and necessary evaluation purposes.

Figure 2a shows the interface of our proposed Raman spectral analysis toolbox, which was developed within the MATLAB R2022a (Mathworks Inc., Asheboro, NC, USA) environment. The software was divided into a set of modules that can accomplish spectral data and regression value loading, spectral preprocessing, data augmentation, model training, model evaluation, and model utilization. The preprocessing module, as shown in Figure 2b, includes the functions of wavelength interpolation, data segmentation, data preprocessing, and data augmentation. In the machine learning module, as shown in Figure 2c, the methods of training the model can be selected for further training and testing tasks, as well as the performance evaluation with statistical indexes. In Figure 2d, it shows the model usage module, including the function of model loading, prediction dataset inputting, monitoring the performance of the prediction model, and the specific prediction results.

### 4.2. Data Loading and Preprocessing

The spectral dataset can be loaded by both *.mat (mat file) and *.txt (text file) formats. For the data in *.mat format, the file should include spectral intensity matrix (*M* × *N*), wavenumber variable (*M* × 1), and a corresponding matrix of spectral regression values (*N* × 1), where *M* represents the number of spectral features and *N* represents the total number of spectra, and therefore each column in the data set represents the observed spectra. The data in *.txt format can be also loaded as multiple files in batches; the text-type spectral data need to include two columns, where one column is the spectral intensity and the other column indicates the wavenumber, but a *.txt file containing regression values, where each spectrum corresponds to the corresponding regression value, is also required. It should be noted that the selected wavenumber range and its interval should be kept in consistent across all spectra.

Obtained Raman spectra will inevitably be affected by environment and instrumental noise, so use of the appropriate preprocessing methods is an essential step to ensure the accuracy of the following regression tasks. In the presented toolbox, the spectral range can be defined by the operator, and a median filter is adapted to eliminate the peak noise caused by cosmic rays. The extended multiplicative signal correction (EMSC) or polynomial fitting is used to subtract the spectral background, and the Raman spectrum is then smoothed with the Savitzky–Golay method. The area normalization of each spectrum after preprocessing could be optionally applied for reducing the influence of instrument and sample changes.

### 4.3. ANN Analysis

#### 4.3.1. CNN

**Figure 8:**
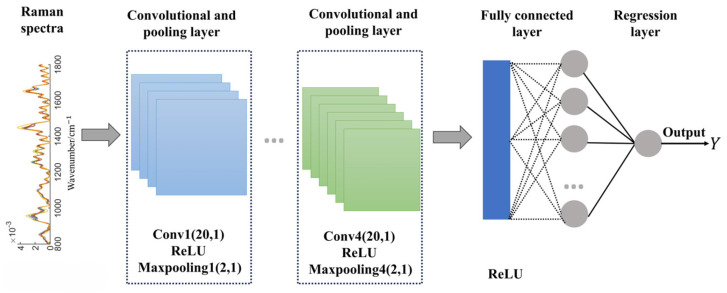
The structure of one-dimensional convolutional neural network, which is incorporated in the presented toolbox.

As a commonly used deep learning algorithm, CNNs are designed to process and analyze data with a grid structure, extensively used in biomedical signal analysis [33,34]. Further, CNNs have been gradually used in quantitative analysis, serving either for direct deployment in regression analysis [35] or for feature selection in regression methodologies [36]. The typical CNN structure includes the convolutional layer, pooling layer, fully connected layer, and activation function. The convolutional layer is the core component of the CNN model, which performs feature extraction from local regions using filters known as convolutional kernels. The pooling layer reduces the data dimensionality while preserving important feature information. The fully connected layer combines features extracted by the convolutional and pooling layers and maps them to the output classes. Activation functions are typically embedded in neurons at each layer to introduce nonlinear properties that enhance the expressive power of the network. The convolutional neural network structure designed in this software is shown in Figure 8. The preprocessed Raman spectra are inputted into a convolutional layer and a maximum pooling layer (repeated four times) for feature extraction. Subsequently, the extracted features are passed to a fully connected layer through a single neuron regression layer for the regression task. A Rectified Linear Unit (ReLU) function is embedded as an activation function in the convolutional layer of the CNN for introducing nonlinearities and thus enhancing the expression and generalization capabilities of the model. Additionally, the learning rate and the number of iterations can be adjusted by the users in the operation panel to improve the performance of the network, as shown in Figure 2c.

#### 4.3.2. MLP Trained with the Backpropagation Algorithm

**Figure 9:**
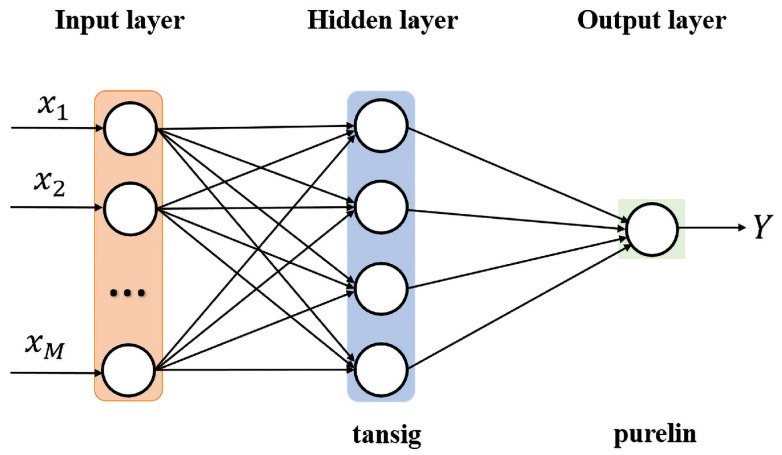
The structure of the backpropagation artificial neural network which is incorporated in the presented toolbox.

An artificial neural network is a computational model composed of multiple artificial neurons that simulates the connections and information transfer between neurons in a biological nervous system. Due to their adaptability, fault tolerance, and nonlinear mapping capabilities, artificial neural networks have been applied in many Raman-related analytical fields. Among them, the MLP (trained with backpropagation) is a widely applied algorithms as a type of multi-layer feedforward neural network, including an input layer, hidden layers, and an output layer [37]. For supervised learning tasks, some form of predictive information is required for model training, including labeled data comprising input features and corresponding target outputs. The BP network learns and stores a model of a great deal of mapping relations of inputs–outputs without disclosing the mathematical equation that describes these mapping relations. Its learning rule is to adopt the gradient descent method in which the backpropagation is used to regulate the weight value and threshold value of the network to achieve the minimum error sum of square. In our presented software, the input data are passed from the input layer of the MLP trained with backpropagation to the hidden layer. In the hidden layer, each neuron calculates a weighted sum and generates an output via an activation function. The output of the hidden layer neurons then serves as the input to the single neuron in the output layer, which computes a weighted sum of these inputs and applies an activation function to produce the final output of the neural network. For this purpose, the “tansig” function is used as a transfer function between the input and hidden layers and the “purelin” function is used between the hidden and output layers in the proposed toolbox, as shown in Figure 9. In addition, the number of neurons in the hidden layer can be changed manually in the interface as shown in Figure 2c and appropriate hyperparameters can be set to improve the network performance and create the desired calibrated model.

#### 4.3.3. Data Augmentation

Although one-dimensional CNN models excel in capturing linear and nonlinear relationships between biological Raman spectra and associated compounds, this deep learning approach demands ample data for effective model training. Practically, it is expensive and difficult to obtain data of some samples due to privacy and other restrictions [38]. In order to make up for various limitations caused by small sample datasets, standard deviation shift was added into the toolbox as a data augmentation method, so as to meet the need of a large amount of data for learning in deep learning. It is also possible to choose whether or not to perform data augmentation when training models using other methods in the toolbox.

### 4.4. Machine Learning Analysis

#### 4.4.1. PLSR

As one of the most commonly used multivariate analysis methods, PLSR integrates multiple linear regression, canonical correlation analysis, and principal component analysis to extract principal components through variable mapping. It effectively addresses the issue of multicollinearity between independent and dependent variables, thereby improving the accuracy, robustness, and practicality of the model [39]. The core idea of the PLSR model is to find a new set of variables that are linear combinations of the original variables, i.e., latent variables. The extraction of these potential variables aims to maximize the covariance between the independent variable and the dependent variable to find the best relationship between them, so as to establish a regression model between the independent variable and the dependent variable. The basic equation of PLSR can be expressed as follows:

(1) Y=X·B+E

where X is the independent variable of the dataset, Y is the dependent variable of the dataset, E is the residual, and B is the regression coefficient matrix for establishing the linear relationship between independent and dependent variables.

#### 4.4.2. SVR

Classification problems involve classifying data into two or more discrete class outputs based on input features, whereas regression problems usually involve predicting continuous predictive values rather than discrete class outputs. Based on the principle of support vector machines (SVMs) [40,41], SVR is characterized by the use of kernels, sparse solutions, and control of the margin and the number of support vectors. Although less popular than SVMs, SVR has been proven to be an effective tool in minimizing the prediction error in a regression task [42]. Without dependence on the dimensionality of input space, SVR establishes relationships between input features and output targets in order to predict continuous values of new input data. Additionally, it has excellent generalization capability, with high prediction accuracy.

Theoretically, SVR is distinct from conventional regression models, like linear regression and random forest regression. For the sample $\left(x_{i},y_{i}\right)$, the general linear model calculates the loss when the model output $g(x_{i})$ is not equal to the real value $y_{i}$, and finally optimizes the model by finding the mean value after gradient descent. However, SVR assumes that the discrepancy between the model output $g(x_{i})$ and the target $\;y_{i}$ is limited to a maximum of *ε*. The loss is calculated only when the absolute difference between $g(x_{i})$ and $y_{i}$ exceeds this threshold. Then, the model is optimized by minimizing the total loss and maximizing the interval. Consequently, the SVR problem can be formulated as the minimization of the following cost function:

(2) $$\psi \left(w,\xi \right)=\frac{1}{2}{\left\|w\right\|}^{2}+C\sum_{i=1}^{n}\left(\xi_{i}+\xi_{i}^{*}\right)$$

subject to

(3) $$\left\{\begin{array}{c} g\left(x_{i}\right)-y_{i}\leq \varepsilon +\xi_{i}^{*}\\ y_{i}-g\left(x_{i}\right)\leq \varepsilon +\xi_{i}\\ \xi_{i},\xi_{i}^{*}\geq 0 \end{array}\right.$$

where w is the weight vector of the model, C is the regularization parameter, and $\xi_{i}\;\mathrm{and}\;\xi_{i}^{*}$ are the slack variables, which are used to measure the deviation of training samples $x_{i}$ outside the intensive zone. In the presented toolbox, two common kernels (linear and Gaussian) can be selected for constructing feasible regression models with the SVR algorithm.

### 4.5. Model Performance Evaluation and Output

To estimate the performance of built regression models, some regression evaluation indexes could be calculated, including SSE, MSE, RMSE, MAE, R^2^, and COR. SSE, MSE, RMSE, and MAE are metrics of the error between the observed and predicted values. R^2^ represents the proportion of variance of response variable interpreted by the explanatory variable [43]. COR stands for Pearson correlation coefficient and measures the linear relationship between predicted and actual values. They can be calculated by the following equations.

(4) $$SSE=\sum_{i=1}^{n}{\left(y_{i}-{\hat{y}}_{i}\right)}^{2}$$

(5) $$MSE=\frac{1}{n}\sum_{i=1}^{n}{\left(y_{i}-{\hat{y}}_{i}\right)}^{2}$$

(6) $$RMSE=\sqrt{\frac{1}{n}\sum_{i=1}^{n}{\left(y_{i}-{\hat{y}}_{i}\right)}^{2}}$$

(7) $$MAE=\frac{1}{n}\sum_{i=1}^{n}\left|y_{i}-{\hat{y}}_{i}\right|$$

(8) $$R^{2}=1-\frac{\sum_{i=1}^{n}{\left(y_{i}-{\hat{y}}_{i}\right)}^{2}}{\sum_{i=1}^{n}{\left(y_{i}-{\bar{y}}_{i}\right)}^{2}}$$

(9) $$COR=\frac{\sum_{i=1}^{n}\left(y_{i}-\bar{y}\right)\left({\hat{y}}_{i}-\bar{\hat{y}}\right)}{\sqrt{\sum_{i=1}^{n}{\left(y_{i}-\bar{y}\right)}^{2}{\left({\hat{y}}_{i}-\bar{\hat{y}}\right)}^{2}}}$$

where n is the number of samples, $y_{i}$ is the corresponding observed value for total n samples, ${\hat{y}}_{i}$ is the predicted value, and $\bar{y}=\frac{1}{n}\sum_{i=1}^{n}y_{i}$, $\bar{\hat{y}}=\frac{1}{n}\sum_{i=1}^{n}{\hat{y}}_{i}$. Theoretically, a regression model with a good predictive performance usually possesses low SSE, RMSE, MSE, and MAE values and high R^2^. Therefore, models with such calculated values in the training and test sets were usually saved for subsequent usage.

### 4.6. Dataset Description

The dataset used in this study is derived from in vivo Raman spectral measurements of human subjects, targeting non-invasive blood glucose monitoring—a typical biomedical application scenario for the AI-Raman toolbox [21,22]. The dataset is constructed based on standardized experimental protocols and rigorous quality control, with detailed information as follows:

#### 4.6.1. Volunteer Information

Number and Health Status: A total of 19 healthy volunteers (12 females, 7 males) participated in the experiment, all of whom were screened to exclude hypoglycemia, diabetes, and other metabolic diseases. The volunteers were aged 20–25 years, with consistent age and metabolic characteristics to reduce inter-individual physiological heterogeneity interference [21,22]. The experiment was approved by the Medical Research Ethics Committee of Northwest University (Approval No. 20220307-2), and all volunteers provided written informed consent prior to participation, in line with international ethical standards for biomedical research.

#### 4.6.2. Spectral Acquisition Details

Raw Dataset Size: After excluding 4 volunteers with unstable spectral signals due to individual physiological heterogeneity, the final raw dataset included 15 volunteers × 7 spectra = 105 spectra. For the toolbox’s functional validation (e.g., small-sample scenario adaptation), a subset of 75 high-quality spectra was selected as the core raw dataset, covering a glucose concentration range of 82.8–180 mg/dL [21,22].

#### 4.6.3. Key Features

Input Features: Each spectrum contains 1024 discrete wavenumber points (800–1800 cm^−1^), forming high-dimensional input features with multicollinearity—consistent with typical Raman spectral data characteristics. Blood glucose concentrations were measured by a Roche ACCU-CHEK Instant fingerstick meter, serving as the reference value for regression model training and evaluation.

### 4.7. Hardware Configuration

All experiments related to spectral data processing, model training, and regression analysis in this study were conducted on a standard desktop computer, representative of common laboratory hardware setups. This configuration was selected to ensure that the AI-Raman toolbox can be run on widely available equipment, avoiding reliance on specialized or high-end hardware (e.g., dedicated GPUs) and thus expanding its accessibility for researchers with limited computational resources. The detailed hardware specifications are as follows:

Central Processing Unit (CPU): Intel Core i7-12700 (12 cores, 20 threads; base frequency: 2.1 GHz, maximum turbo frequency: 4.9 GHz). This CPU provides enough computational power for the toolbox’s integrated algorithms, including the training of deep learning models (e.g., CNNs) without relying on GPU acceleration.

Random Access Memory (RAM): 16 GB.

Software Environment: Windows 10 Professional (64-bit) with the MATLAB R2022a environment (the development platform of the AI-Raman toolbox).

## Data Availability

This Raman spectra analysis software is open source and can be freely available at https://github.com/wsnwuphy/AI-Assisted-Raman-Spectra-Analysis-Toolbox (accessed on 30 December 2025. Software name: AI-Raman (AI-Assisted Raman Spectra Analysis Toolbox).

## Abbreviations

The following abbreviations are used in this manuscript:

RS	Raman spectroscopy
PCA	Principal component analysis
LDA	Linear discriminant analysis
QDA	Quadratic discriminant analysis
PLS	Partial least squares
DA	Discriminant analysis
ANNs	Artificial neural networks
CNN	Convolutional neural network
BP	Back propagation
GA	Genetic algorithms
PLSR	Partial least squares regression
SVR	Support vector regression
SSE	Sum of squares due to error
MSE	Mean squared error
RMSE	Root mean squared error
MAE	Mean absolute error
COR	Pearson correlation coefficient
EMSC	Extended multiplicative signal correction
ReLU	Rectified Linear Unit
SVM	Support vector machines
MLP	Multi-Layer Perceptron
POG	Post oral glucose
CPU	Central Processing Unit
RAM	Random Access Memory
